# Forensic Psychiatry

**DOI:** 10.1192/j.eurpsy.2022.62

**Published:** 2022-09-01

**Authors:** B. Völlm

**Affiliations:** University Medicine Rostock, Forensic Psychiatry, Rostock, Germany

## Abstract

In this session I will discuss recent publications that have advanced the field of forensic psychiatry or changed clinical practice. Content will be current so that it is not possible to specify at present.

**Disclosure:**

No significant relationships.

